# Global mental health and neuroethics

**DOI:** 10.1186/s12916-015-0274-y

**Published:** 2015-03-04

**Authors:** Dan J Stein, James Giordano

**Affiliations:** Department of Psychiatry & MRC Unit on Anxiety & Stress Disorders, University of Cape Town, Groote Schuur Hospital, Rondebosch, Cape Town, 7700 South Africa; Neuroethics Studies Program, Pellegrino Center for Clinical Bioethics and Department of Neurology, Georgetown University Medical Center, Washington, DC USA; Human Science Center, Ludwig-Maximilians Universität, Professor-Huber-Platz 2, 80539 München, Germany

**Keywords:** Global mental health, Neuroethics, Psychiatry, Neuroscience

## Abstract

**Background:**

Global mental health is a relatively new field that has focused on disparities in mental health services across different settings, and on innovative ways to provide feasible, acceptable, and effective services in poorly-resourced settings. Neuroethics, too, is a relatively new field, lying at the intersection of bioethics and neuroscience; it has studied the implications of neuroscientific findings for age-old questions in philosophy, as well as questions about the ethics of novel neuroscientific methods and interventions.

**Discussion:**

In this essay, we address a number of issues that lie at the intersection of these two fields: an emphasis on a naturalist and empirical position, a concern with both disease and wellness, the importance of human rights in neuropsychiatric care, and the value of social inclusion and patient empowerment.

**Summary:**

These different disciplines share a number of perspectives, and future dialogue between the two should be encouraged.

## Background

Global mental health is a relatively new but highly influential field that has emerged from an awareness that there is ‘no health without mental health’ [1] and that there is a need for psychiatric care that is not only cross-culturally appropriate [2,3] but that can also be scaled up across the globe [4]. Global mental health has relied on a number of pre-existing fields of study. First, cross-national epidemiological studies have emphasized that mental disorders are prevalent across the globe, but are underdiagnosed and undertreated, with the treatment gap widest in low- and middle-income countries [5]. Second, studies in psychiatric anthropology, including work with immigrants and refugees, have emphasised that understanding of and interventions for mental disorders need to include an appreciation of the relevant socio-cultural context [6]. Practitioners of global mental health have focused on advocating for measures to close the treatment gap and on developing novel ways of doing so in under-resourced regions [7], and such work has already had a significant impact on psychiatric research and practice.

Neuroethics is similarly a relatively new but highly influential field, which has emerged from ongoing work in the areas of neuroscience, psychiatry, neurology and bioethics, and that is becoming increasingly international in scope [3,8,9]. On the one hand, neuroethics poses the question of whether and how new findings in basic and clinical neuroscience shed light on long-standing questions in philosophy, including the relationship between brain and mind, and the nature of morality (that is, the ‘neuroscience of ethics’) [10]. On the other hand, neuroethics has addressed the ethical questions fostered by novel neuroscientific methods and their applications in research and medicine, including functional brain imaging, neurogenetic screening, psychopharmacological treatments and enhancements, and the social implications of various neurotechnological interventions, such as deep brain and transcranial magnetic stimulation (that is, the ‘ethics of neuroscience’) [11]. Thus the field draws on, but also expands bioethical work, and has given rise to a rich set of interdisciplinary writings that cover a broad range of issues [12].

While the broader convergences between psychiatry and neuroethics also deserve attention, in this essay we argue that some of the intersections between the important fields of global mental health and neuroethics are particularly timely and potentially fruitful. The foci of the neuroethics of global mental health are likely to range from long-standing questions in psychiatric ethics, to more recent issues that have emerged as a result of relatively new developments in the applications of neuroscientific methods to mental health research and care. We will discuss in turn how both global mental health and neuroethics have an emphasis on a naturalist and empirical approach, on both disease and wellness, on human rights in neuropsychiatric care, and on the value of social inclusion and patient empowerment (Table 1). Indeed, while global mental health and neuroethics are quite different disciplines, they share a number of important perspectives, and an ongoing dialogue between them should be encouraged.

**Table 1:** **Intersections between global mental health and neuroethics**

**Area of Intersection**	**Global mental health**	**Neuroethics**
Naturalist and empirical approach	* Importance of evidence-based clinical practice locally and globally	* Advances in neuroscience may shed light on broad philosophical questions
	* Particular need for mental health research in low- and middle-income countries	* Value of empirical approaches to answering specific bioethical questions
Concern with both disease and wellness	* Focus on absence of disease as well physical, mental and social well-being	* Particular interest in the possible value of neuro-enhancement
	* Emphasis not only on symptom reduction but also on recovery	* Commitment to using technologies to maximise potential of all
Importance of human rights in neuropsychiatric care	* Emphasis on the human rights of those suffering from mental illness	* Emphasis on the social and legal implications of neuroscientific advances
	* Importance of equivalent prioritization of mental and physical health	* Concern that neurotechnologies may fortify asymmetric relationships
Value of social inclusion and patient empowerment	* Emphasis on value of consumer perspective; ‘nothing for us, without us’	* Role, relevance, and importance of brain science to concepts of ‘self’
	* Focus on establishing and enhancing patient empowerment	* Emphasis on the meaning of neuroscience, and its contribution to flourishing

### Evidence-based medicine/empirical neuroethics

As a discipline, global mental health has emphasised the importance of evidence-based clinical practices [13]. Of particular relevance to attempts at employing evidence-based medicine (EBM) in global mental health is the 90:10 research gap: the vast proportion of mental health research (90%) has focused on the relatively small proportion of the world’s population that lives in high income countries (10%) [14]. Likewise, clinical neuroscience research has focused primarily on westernised, educated, industrialised, rich and democratic (WEIRD) populations [15]. Thus, there is a clear need for additional mental health research to be undertaken in low- and middle-income countries.

The need for empirically defined and articulated research is equally crucial to neuroethics. A key pillar of the field is a naturalistic view that posits that advances in neuroscience may well shed light on philosophical issues [16-18]. For example, the field has emphasised the importance of neuroscientific approaches to examining fundamental questions about the nature of the self, agency and responsibility, noting how novel findings about the neuroanatomy, neurophysiology and neurogenetics of decision-making and impulse control influence current understanding of these constructs. Neuroethics has also been particularly focused upon empirical approaches to bioethical questions and has obtained data on a broad range of such issues [18]. For example, advances in neuroimaging have provided impetus to exploring empirical questions in neuroethics (such as whether imaging findings in psychiatric disorders increase or decrease stigma? [19]); but with the introduction of each novel instrument or approach new queries and issues arise as to the validity and value of these tools in research and clinical practices.

The emphasis of global mental health on evidence-based practice and of neuroethics on empirical ethics, are not entirely without controversy. It has often been pointed out that an absence of evidence does not always reflect evidence of absent efficacy or effect [20]. Given that most mental health research has been undertaken in areas where only a minority of the population lives, it must be acknowledged that straightforward extrapolation of the existing evidence-base is not always appropriate. For example, there is a paucity of psychotherapy trials conducted in the low- and middle-income world, so that the extent to which Western psychotherapies require adaptation in such contexts is somewhat unclear. In addition, there may well be a need for value-based orientations to supplement EBM [21]. Within meta-philosophy, there are strong arguments that philosophy should not simply be reduced to science [22]. Similarly, in bioethics, there may well remain complex problems that are best apprehended by conceptual rather than empirical analysis.

Nevertheless, there are good reasons to support an empirical approach in both global mental health and neuroethics. Global mental health has emphasised that precisely because so much work has been done in areas where only a minority live, it is important to expand research efforts in the low- and middle-income world. Priorities for such research have been carefully set, and this has facilitated requests for proposals to fund such research [23]. In neuroethics, empirical research has contributed to a range of discussions, including work on childhood development, ageing research, the use and value of various neurotechnologies, as well as how neuroscientific and psychosocial approaches can be employed to develop improved assessment and treatment of mental, neurological, and substance use disorders [11].

### Focus on both disorder and wellness

The World Health Organization (WHO) definition of health encompasses the concept of wellness, noting that health is ‘a state of complete physical, mental and social well-being and not merely the absence of disease or infirmity’. This focus is consistent with the emphasis in global mental health on human rights to access healthcare, and on patient empowerment [1], and we can expect that global mental health will pay increasing attention not only to disorders but also to well-being. Clinical trials of task-shifting of mental health interventions to lay community health workers in low- and middle-income countries, for example, may well include outcomes designed to measure both symptom severity and also patient recovery [24].

There is ongoing neuroethical discussion about whether the introduction of new neurotechnologies for the assessment and treatment of neuropsychiatric disorders should also be used in those with sub-threshold symptoms, or to exceed ordinary capacities [25]. While part of the neuroethical focus is to ensure that novel technologies are appropriately developed and correctly used, another part reflects a commitment to employing such technologies in ways that maximise the potential (and relative) benefit for individuals and for societies [17].

There are, however, clear concerns with an emphasis on well-being. While there is some agreement on what constitutes a typical physical or mental disorder (for example, a severe acute infection), there are persistent ambiguities about the boundaries of normality (for example, should binge-drinking be considered a mental disorder?) [8], and there is even more disagreement about what constitutes ‘well-being’ and flourishing (for example, some definitions of well-being include notions of ‘career consolidation’, which may not be equally relevant in all parts of the globe) [26]. Furthermore, while there are relatively good data on the efficacy and cost-effectiveness of certain interventions for psychiatric disorders (for example, antidepressants for severe depression), there is a paucity of data on the efficacy and effectiveness of interventions for ‘well-being’ [26]. To a great extent, such criteria and definitions may be cultural, and Parens [27] and Sandel [28] have written persuasively about the problem of relying upon both Western constructs of, and technological approaches for addressing and attempting to resolve, social ills.

It is our view that such controversies surrounding the concept and/or conceptualisations of well-being and flourishing demand and deserve attention. Both global mental health and neuroethics have been critical of a reductionistic approach to disorder [29,30]. Global mental health is likely to focus initial research efforts on the prevention and treatment of serious mental disorders, rather than on maximising well-being in the community [23], but even in this work it will be important to address and emphasise patient recovery and empowerment. Similarly, while neuroethics will seek to carefully monitor and guide the appropriate use and just provision of new neurotechnologies, novel opportunities to improve individual and societal flourishing will become an increasingly prominent topic for debate and practice [17].

### Human rights/parity for mental health

Global mental health has placed significant emphasis on acknowledging the human rights of those suffering from mental illness [31]. A fundamental premise in this discourse is the right to appropriate treatment; there is ‘no health without mental health’ [1] and any meaningful approach to ‘global health’ must entail parity in resourcing for physical and mental health services. Mental health policies are required that ensure mental health and medical services are equivalently prioritised, and which indicate how the human rights of those suffering from mental disorders will be vouchsafed during the development of such services. Such policies should ensure that mental health will be integrated into health services, and that these will be made available in community settings.

Neuroethics, too, has been concerned with broad issues of social policy. Indeed, the intersection of neuroscience and social policy has been characterised as one of the four pillars of neuroethics [16], concerned with the social and legal implications of neuroscientific advances, including health care disparities, and unequal access to the benefits of such advances. For example, in considering issues of neuroenhancement, it has been argued that social resources should be conserved for treatment (rather than enhancement), and that enhancement (for example, the use of stimulant medications by students and professionals to increase performance) may unfairly favour more privileged sections of society that can afford such interventions [28]. Therefore, it is important to address if, and ensure that, neurotechnologies are not inappropriately used or purposefully misused to fortify asymmetrical relationships between individuals, groups and nations [8].

Even so, a focus on human rights and mental illness is not without controversy. First, concepts of human rights, and indeed the idea of human rights itself may not represent a fixed natural kind, but rather may be bound to specific times and places [32]. In light of this, a good deal of conceptual work may be required in order to argue for specific universal human rights. Further, the range of moral concepts that can be employed to understand and evaluate an ethical issue goes far beyond the class of rights; such additional moral concepts include ‘duty’, ‘the good’ and ‘virtue’ [33]. This point again emphasises the need for additional conceptual analysis in this area.

Nevertheless, emphasising that the need for appropriate treatment of mental disorders is closely linked to human rights has provided global mental health with an important ethical foundation. Similarly, there is an ongoing need to emphasise human rights in the conduct of neuroscience, so as to sustain ethical practices in basic and clinical neuroscientific research, and sound ethical precepts in establishing and implementing clinical assessments and interventions. Such concerns constitute a second pillar of neuroethics [12,16]. As the agenda of global mental health gives increasing impetus to mental health research being undertaken in low- and middle-income countries [23], so, too, will there be a need to concomitantly increase attention and dedication to neuroethical issues arising in and from these settings [17].

### Social inclusion/consumer movements

A recurring rallying cry in global mental health has been ‘nothing for us, without us’, emphasising the importance of including consumers in decision-making about services and research [34]. The voice of the consumer movement has played a crucial role in disability studies in general and is particularly relevant to mental health concerns [35]. Global mental health publications have emphasised the importance of consulting consumers and the value of a ‘recovery perspective’ that seeks to establish and enhance patient empowerment [24]. Similarly, much of the literature in global mental health has emphasised the importance of ensuring that assessments and interventions developed for use in low- and middle-income countries are, in fact, feasible and acceptable to individuals who live in these contexts [36].

Likewise, neuroethics has acknowledged the importance of individuals’ subjective experiences and of patient empowerment. Brain imaging, for example, provides certain objective correlates of human cognition and affect. Yet, imaging data cannot reveal or ‘describe’ an individual’s unique thoughts and emotions [18]. A gap persists between subjectivity and objectivity, which is important to acknowledge and address if, and when, considering the utility and limitations of specific neuroscientific approaches to diagnostics and clinical intervention. A third pillar of neuroethics has focused upon the role, relevance, and importance of brain science to concepts of the ‘self’ [12,16]; for instance, data that certain genetic variants are associated with decreased impulse control may lead to more nuanced discussion of the nature of moral responsibility and free will. Similarly, literature on psychopharmacology has discussed issues such as the meaning of medication for patients, the extent to which the self is transformed by pharmacotherapy, and the extent to which psychotropic agents not only improve symptom outcomes but may also contribute to human flourishing [37].

Still, an emphasis on patients’ subjective experience and empowerment is not without controversy. Robert Spitzer, a key architect of the *Diagnostic and Statistical Manual of Mental Disorders, third edition* (*DSM-III*), argued that including patients when revising psychiatric nosology would be ‘…political correctness taken too far’ [38]. Advances in neuroscience seem to promise a world in which clinical diagnosis will be made using biomarkers, neuroimaging and neurogenetics, rather than by merely assessing patients’ history and symptoms [39]. To be sure, neurobiologically-based models (for example, that are reliant upon and derived from neurogenetics, neuroanatomy and/or neurochemistry) of mental disorder(s) may well have important advantages to the extent that neurotechnologies, such as brain imaging and biomarker analyses, may enable more accurate diagnosis and effective treatment [40,41], provided that potential burdens and harms are minimised [8,12,17].

Nevertheless, an emphasis on social inclusion, consumer voices and patient empowerment is sure to remain important for global mental health, neuroethics and their intersection. The global mental health movement is likely to pay particular attention to any suggestion that it is ignoring such issues [42]. Neuroethics has a strong commitment to proactive and preparatory stances that promote both responsible social and public policies, and the value of empowerment [18,43]. Moreover, in mental health and neuroethics – as in medicine, in general - there is a need for an integrative, conceptual approach that addresses biological mechanisms that underlie the symptoms of mental disorders, as well as patients’ expression and experience of such symptoms [12,37].

## Summary

Global mental health and neuroethics are two important fields of study and practice which, while coming of age over the past decade, have had relatively little direct interaction. Perhaps this is not surprising given the differences in their foci. Global mental health has focused upon the low- and middle-income world, while neuroethics has tended to address issues somewhat more relevant to Western, higher income countries. Global mental health is focused on implementation science - often adapting well-studied interventions to new contexts, while neuroethics is focused on new technologies and novel techniques. Global mental health has emphasised the value of employing lay community health workers to assist with mental health interventions, while neuroethics has explored highly specialised technical procedures such as functional brain imaging and deep brain stimulation.

Yet, as these disciplines mature, consolidate, and are joined by the growing foci and applications of social and cultural neuroscience [44], and expanding efforts in global mobile health (mhealth) and in neurogenetics [45,46], we can expect that there will be a number of important opportunities for dialogue and mutual engagement in practice. Our view is that there are a number of areas in global mental health and neuroethics that foster and support converging perspectives, including an emphasis on a naturalist and empirical position, a concern with both disease and wellness, the importance of human rights in neuropsychiatric care, and the value of social inclusion and patient empowerment (Table 1). While there are also important intersections between psychiatry as a whole and neuroethics, these convergences between global mental health and neuroethics seem to have particular potential for growth and for positive social effects.

The differences and convergences between global mental health and neuroethics may well lead to a rich conversation between these fields. This essay, albeit brief, may contribute a small start; we look forward to a more extended and detailed discussion. Such work will hopefully address a range of reciprocally pertinent issues, including the neuroethical aspects of current global mental health research, as well as the expansion of neuroethical research to address the probity of allocating new developments in neuroscience and neurotechnology to the low- and middle-income world. Our view and aspiration is that just as psychiatric research in low- and middle-income countries may have lessons for high-income countries, so too lessons from basic, clinical and cultural neuroscience in non-WEIRD settings may impact positively on the development and articulation of an international neuroethics that is capable of guiding neuroscience and its clinical applications throughout the globe.
